# Expanding Notions of LGBTQ+

**DOI:** 10.1146/annurev-soc-030320-032256

**Published:** 2023-03-31

**Authors:** Stephen T. Russell, Meg D. Bishop, Jessica N. Fish

**Affiliations:** 1 Department of Human Development and Family Sciences, Population Research Center, University of Texas at Austin, Austin, Texas, USA; 2 Department of Family Science, School of Public Health, University of Maryland, College Park, Maryland, USA

**Keywords:** sexual minority, LGBTQ, sexual identity, gender identity, intersectionality, life course

## Abstract

Sexual identity labels and meanings have been expanding. We explore how sexual identities are taking shape, intertwining, and emerging in new forms among a growing number of LGBTQ+ people (i.e., lesbian, gay, bisexual, transgender, queer, and questioning, or people whose identities are outside the historically privileged or dominant groups of heterosexual sexual identities). We situate contemporary sexual identities in theories of the social construction of identity, intersectionality, and the life course. We review recent research that illuminates identity complexity and intersectionality, the increasingly intertwined understandings and experiences of sexuality and gender, and intersections of sexuality and gender with identities embedded in race and social class. Finally, we consider new work that situates sexual identities in the context of life course development, including life stage, developmental processes, and relationships.

## INTRODUCTION

Sexual identities, both social and personal, evolve across sociohistorical time; this has been particularly true in the past decade. Sexual minority and gender minority lives and identities have been understood as distinct yet intertwined. Indeed, sexual and gender minority identities and communities are often described together—as lesbian, gay, bisexual, transgender, queer, questioning, and other emerging sexual (and gender) identities (LGBTQ+). In this review, we consider a primary aspect of that change, expanding notions of LGBTQ+—or how sexual identities are taking shape, intertwining, and emerging in new forms among a growing number of people who fall under the LGBTQ+ umbrella, or outside the historically privileged/dominant groups of heterosexual sexual identities. We trace expanding notions of LGBTQ+ identities to growing visibility and integration of LGBTQ+ people and issues in the United States and around the world, and associated growing numbers of people for whom the LGBTQ+ umbrella resonates as salient for personal identity.

Sexual identity has been conceptualized in reference to one’s own and a (potential) partner’s sex or gender; sexual identity refers to identity based on physical or romantic attraction and includes identity based on desires or behaviors and expressed in sexual identity labels (or lack thereof). (Gender identity, considered further below, reflects one’s sense of being a man, a woman, both, or neither, and it has typically been conceptualized as distinct from sexual orientation and identity; see the glossary of terms in Table 1.) Central to understanding expanding sexual identities is the concept of stigma. In their influential *Annual Review of Sociology* article, Link & Phelan (2001) conceptualized stigma as constituted through the co-occurrence of several components: labeling of differences, stereotyping based on those differences, categorization and separation of in- and out-groups, and status loss and discrimination which produce inequalities (Link & Phelan 2001, Natl. Acad. Sci. Eng. Med. 2020). Thus, sexual identities come from or are defined by stigma that shapes social and interpersonal interactions and, ultimately, individual self-labels and meanings.

The rise of new media and global communication has meant that diversity of sexual identity is more accessible and visible than ever. As people increasingly share personal experiences and understandings of the self beyond their immediate in-person networks (Craig & McInroy 2014), notions of sexual identity become increasingly permeable: More communities and cultures can access and, in turn, shape possibilities for sexual identities. Growing numbers of people feel comfortable questioning or exploring possibilities for their sexual identity. Increasingly, people have come to see their experiences of sexuality (and gender) outside of historically typical or dominant sexual identities (lesbian, gay, bisexual, and heterosexual). For growing numbers, there are more opportunities to question the historic binaries of sexuality (straight or gay) and gender (man or woman), which, for many, resonate as limiting of lived experience. New identity terms have emerged, providing language for experiences that had previously been unnamed. These new articulations of identities and experiences can be shared more rapidly and with new reach, including the category of those who reject identity labels for describing or understanding sexuality and identity in the first place (Bates et al. 2020).

In the review that follows, we explore contemporary sexual identities, drawing from theories of the social construction of identity, intersectionality, and the life course. Personal identities emerge through social interactions and interpretations of shared experience. Importantly, stigma related to sexuality and gender defines norms and dominance, creating categories of what is normal or consistent with dominant and oppressive expectations. Thus, identity and experience are constituted from multiple vantage points and experiences that are inseparable, or intersectional, particularly with respect to marginalized social positions. Furthermore, who we are and how we understand ourselves are situated both historically and ontologically, developmentally, and interpersonally, in the context of the life course. These frameworks illuminate social change and the expanding notions of contemporary sexual identity. As such, we consider identity complexity and intersectionality, including the increasingly intertwined understandings and experiences of sexuality and gender, as well as the intersections of sexuality and gender with identities embedded in racialized identity and social class. Finally, we reflect on life course development, including the salience of life stage, as well as developmental and relational dimensions of contemporary sexual and gender identities.

## PERSONAL YET SOCIAL IDENTITIES

Theoretical foundations of identity from sociology and psychology provide grounding for understanding expanding notions of LGBTQ+. Hammack (2015, p. 11) describes identity as the “anchoring concept” for understanding difference and sameness in contemporary times. Hammack characterizes identity as a concept that has meaning far beyond the theoretical complexities of academic literatures and has permeated everyday conversations and the ways we understand ourselves in the world.

George Herbert Mead’s (1934) symbolic interactionist conceptualization of identity emphasizes the dynamic between a person’s social interactions and their internal understanding of the self. In this dynamic, social interactions are the basis of understanding the self as well as the development of—and affiliation with—social categories (Hammack 2015). Personal or collective understanding of and alignment with social categories are the basis of identities. These foundational concepts of the social interactional or relational perspective on identities point to several dimensions that are relevant for understanding contemporary sexual identities: the role of stigma, the meaning of labels, the developmental nature of identities, and the meaning and interpretations of queer theory and experience. But identities are not simply social categories or labels; they are also the symbols for the image we have of ourselves in the social world (Owens et al. 2010). This understanding of the meanings of labels—as they emerge in social interactions and are claimed by individuals—is foundational for understanding the expansion of sexual identity labels under consideration, given that relatively greater accessibility of sexual diversity has provided a foundation for both social and intrapersonal expansions of LGBTQ+ identity.

Identities shift in nature and character across time and culture. For individuals, personal identities may develop or change. Historically and culturally, the meaning ascribed to an identity may shift across time and place, including possibilities for new identities to emerge (Howard 2000, Owens et al. 2010). Erikson’s (1968) foundational conceptualization of identity was explicitly developmental: That is, developing and solidifying one’s identity are a core task of human development and are central in the developmental period of adolescence. Adolescence is understood as a crucial period of exploration with respect to one’s sense of self, including one’s place in the social world. Importantly, this conceptualization viewed identity not only in terms of an individual’s sense of self. For Erikson, identity was also central to the process through which people coconstruct the social order. With respect to contemporary sexual identities, the relevance here is to understand how identities are individually meaningful, coconstructed in social context and with social implications, and developing across time, in terms of both individual human development and changing social meanings.

Finally, queer theory and experience have offered one of the profound societal shifts in the personal and social meanings of identity. Queer theory illuminates the ways that sexuality cannot be understood in terms of binary categories (of homosexual versus heterosexual) and, further, that binaries of sexuality (as well as gender and other binaries) are dynamic or intertwined and are defined in terms of power (Gamson & Moon 2004). By challenging binary boundaries, queer perspectives also blur the distinction between sexuality and gender. For example, a genderqueer identity may be not solely a sexual identity or a gender identity, but an identity that, for some, is fundamentally at the intersection of queer sexuality and gender (Barsigian et al. 2020). Furthermore, queer identities challenge binaries beyond sexuality/gender, including other identity statuses that constitute the self, such as racialized identity, ethnicity, social class, or religion (Gamson & Moon 2004). For example, “Gaysian” is a contemporary identity that acknowledges the inseparability of being Asian American from being LGBTQ+ (Austin 2016, Eguchi 2020). What these examples point to are the ways that notions of LGBTQ+ are expanding into new—and more—labels for identities that characterize sexuality as integrated into the diversity of personal and social identities.

## INTERSECTIONALITY

Originating from late-twentieth-century Black feminist movements, intersectionality theory (Cohambee River Collective 1977, Collins 2015, Crenshaw 1991, Else-Quest & Hyde 2016a, McCall 2005) describes how multiple and interlocking forms of structural privilege and oppression, such as sexism, racism, and classism, intertwine to shape experiences of those who hold multiple marginalized identities across race, gender, class, and sexual identity, among others. Thinking intersectionally about sexual minority identities therefore requires the conceptual understanding that identities are mutually constituted at intersecting social positions, and it calls for empirical approaches that use methods that capture and conceptualize such intersecting social positions.

In the past decade, intersectionality research attending to sexual minority identity has been focused primarily on structural intersectionality, which explores how intersecting social identities converge to structure and distinguish experiences of privilege and oppression (see Crenshaw 1991). For example, many recent studies have used intercategorical approaches (see McCall 2005) to compare subgroups at the intersections of sexual identity and other social identities, such as gender, marital status, and family composition, in the contexts of physical health, romantic relationships, and child health. In their examination of how sexual orientation and gender intersect to influence self-rated health, Gorman et al. (2015) showed that bisexual men and women reported the poorest health relative to other groups organized by gender and sexual identity. Upon closer investigation, the authors observed that bisexual participants were disproportionately disadvantaged on social, behavioral, and economic factors (e.g., lower socioeconomic status, less medical insurance), indicating the health-compromising influences of multiple marginalized identities at such intersections. In a similar investigation, Solazzo et al. (2020) found that among sexual minority people, the best health status was found among those who were married; however, bisexual people in the sample, particularly women who were formerly married, reported more health and socioeconomic disadvantage than other subgroups. In another examination of the stability of same- and different-sex relationships, Joyner et al. (2017) found that male couples had significantly higher rates of dissolution from the time that the relationship formed than female or different-sex couples; however, female same-sex couples had the highest dissolution rates when authors examined dissolution starting from the period of coresidence.

Intracategorical intersectional research focuses specifically on one often traditionally marginalized social group in order to delve deeply into the complexity of the group’s lived experiences (McCall 2005). Moore’s (2012) work on Black gay women forming families describes the means by which race and racism, in particular, shape lived experiences (see also Moore & Stambolis-Ruhstorfer 2013). Moore contends that experiences with racism shape fundamentally distinct goals for Black lesbian women’s motherhood. Moore describes a unique tension that Black women must navigate, maintaining their status as respectable within Black communities while also navigating sexual autonomy and meeting their familial goals as lesbian women. In *Men in Place*, Abelson (2019) recounts the experiences of trans men across the US Midwest, South, and West. The author describes how trans men are impelled to adopt a “regular guy” role and how this persona varies across distinct social locations of sexual identity, racialized identity, class, and place. Specifically, Abelson details the ways in which trans men with relatively more privileged intersectional identities (e.g., upper-class, urban White men) are allowed more access to the narrative of the regular guy in less urban and resourced locations, highlighting the ways in which place, race, and sexuality dictate acknowledgment of masculinity.

Additional intracategorical studies focus on the intersectional nature of stigma and discrimination within certain subgroups of sexual minority people. For example, Mallory & Russell (2021) examined how multiple forms of discrimination, including racism and homophobia, operate to influence mental health in a sample of sexual minority youth of color. Their findings suggested that homophobic discrimination and racial discrimination intersected to compound their negative influences on depression, while these forms of discrimination independently influenced risk for suicidality. Such approaches highlight recent calls from intersectionality scholars (Bauer & Scheim 2019, Bowleg & Bauer 2016, Lewis 2017, Mallory & Russell 2021, Toomey et al. 2017) to prioritize the processes of power and inequality that dictate experiences at the intersections of social location (e.g., racism, homophobia, sexism), as well as to operationalize intersecting identities.

Several recent theoretical pieces and commentaries (Aguayo-Romero 2021, Bowleg 2021, Moradi & Grzanka 2017) have described the flattening of intersectionality as the theory becomes more mainstream—more specifically, they problematize the notion that intersectionality is equivalent to measuring multiple identities, and they emphasize the need to center power and inequality. As sexual minority identities shift, expand, and diversify, understanding the ways that they relate to intersectional systems of power and privilege (e.g., heteronormativity, cisnormativity, homophobia), rather than simply to new categorizations of sexual minority people, will be an important contribution.

## THE LIFE COURSE

Life course theory provides a valuable framework for understanding sexual diversity in the context of individual development, relationships, and society. This perspective offers a dynamic view of people’s development and interdependence across time and place. Furthermore, it considers how individuals are agentic in shaping social structure inasmuch as they are shaped by it (Roy & Settersten 2023). The growing scientific and public attention to sexual (and gender) diversity reflects shifts in social structure and societal values, including social and policy backlash, in the context of changing LGBTQ+ authenticity and visibility.

Life course perspectives are often cited as an essential framework for understanding sexual minority populations (Natl. Acad. Sci. Eng. Med. 2020); however, their use in the scholarship of sexual minority people and sexual diversity is inconspicuous (Hammack et al. 2018, Russell & Fish 2019). Fundamentally, a life course perspective is rooted in overlapping sociogenic and ontogenic change and is concerned with the social contexts and influences of human development across life stages—with specific attention to time and place. Life course theorists reflect on the importance within context of human agency, linked lives, and the timing of life events as key features of understanding trajectories of human development, lived experience, and related outcomes (e.g., relationships, health).

Given the emphasis on contextual influences of development, life course theory views the structure of people’s lives as guided by social institutions and policies, which emphasize normative pathways of development, including sexual normativities (e.g., heteronormativity) (Carpenter 2010). Although people have agency in their development and experiences within context, the navigation of these social institutions and the practices therein typically fall within the bounds of what is normative, referred to as “agency within structure” (Settersten & Gannon 2005). Still, given agency and social change and the interactions therein, destandardizations of the life course occur, in which individuals and, in some instances, cohorts reflect heterogeneity, deviation, and diversity (Roy & Settersten 2023). These instances of destandardization also provide the basis for understanding marginalization, inequity, and the cumulative disadvantages that shape the lived experiences of sexual minority people as they push back on concepts of normativity of sexuality, gender, and family across the life course, particularly in previous decades (Oswald et al. 2005, 2009).

Within one generation, sexual minority people in the United States experienced dramatic social, political, and legal changes, from pathologization and criminalization to widespread legal and (ostensibly) social acceptance (Hammack et al. 2018). These markers of social change include the Stonewall riots of 1969, the removal of “homosexuality” from the *Diagnostic and Statistical Manual of Mental Disorders*, the AIDS epidemic in the 1980s, the decriminalization of same-sex sexuality through Lawrence v. Texas (2003), marriage equality through Obergefell v. Hodges (2015), and workplace protections via Bostock v. Clayton County (2020). Comparatively, more recent policies reflect a backlash against social progress, including the introduction of Florida’s “don’t say gay” bill in 2022 and the deluge of anti-trans policies seeking to limit transgender youth sports involvement, ban gender-affirming care, and criminalize physicians and families who provide trans-affirmative mental and medical health services (Fish & Russell 2022). Although this progress has not been linear (e.g., the 1996 Defense of Marriage Act, Pub. L. 104–199, 110 Stat. 2419) and is heterogeneous across regions and states (e.g., “conversion therapy” bans), these social changes uniquely shape and demarcate the developmental experiences of sexual minority people of different cohorts and generations (Hammack et al. 2018).

Perhaps the clearest example of the changing meanings of sexual identities across historical time and cohorts is the dynamic tension between growing social acceptance for sexual minority people (McCarthy 2021, Pew Res. Cent. 2019) and the declining age of sexual identity disclosure across cohorts of sexual and gender minority people in the United States (Bishop et al. 2020, Martos et al. 2015, Puckett et al. 2022). Visibility and social change have created the possibility for sexual minority people to understand themselves as sexually diverse and “come out” at earlier stages of the life course (Russell & Fish 2019). Still, despite social progress for sexual minority populations, greater visibility and earlier sexual identity awareness and disclosure reflect destandardizations from the typical life course and continue to receive varying degrees of implicit, overt, and structural stigma. In fact, this timing of disclosure in the life course, within this historical moment, creates a developmental collision between ontogenetic and sociogenic development for the current cohort of sexual minority youth. Specifically, sexually diverse youth now come out in adolescence, a time of unique developmental characteristics, including heightened self-consciousness, peer conformity, higher rates of victimization, and more social and self-regulation, particularly around sexuality and gender (Fish 2020; Russell & Fish 2016, 2019). Furthermore, sexual minority people who come out at younger ages are more likely to be financially dependent on family, which may jeopardize their emotional and physical safety in instances where families are not accepting. These developmentally situated stressors then heighten the risk for poor developmental outcomes.

## EMERGING LABELS, EXPANDING IDENTITIES

We have suggested that to best understand expanding notions of LGBTQ+, we must understand the social construction of identities in ways that account for their intersectional meanings and the ways that they are constructed in life course time and place—that is, in terms of both changing sociohistorical times and individual development. We now turn to a discussion of research that describes and illuminates new and changing meanings and possibilities for contemporary identities and labels. First and fundamentally, understandings and experiences of sexuality and gender are intertwined: While sexuality and gender remain distinct yet intersecting identity dimensions, for a growing number of people the boundaries have become blurry in ways that redefine identities. These intersections of sexuality and gender are, of course, embedded in racialized, ethnic, and social class identities. Finally, we reflect on intersectional sexual identities in the context of life course development, including the relevance of life stages and relationships. Here, life stage is especially crucial, as historical change intersects with age cohorts in ways that have produced opportunities for new understandings and experiences of sexual identities.

### Complexities of Sexuality and Gender

Sexual identity has been conceptualized in reference to one’s own and a (potential) partner’s sex or gender, and gender identity typically has been conceptualized as distinct from sexual orientation and identity. Clearly, these distinctions remain relevant, yet more and more people, particularly in younger cohorts, claim sexual identities or labels that not only blur the binaries of sexuality (gay or straight) and gender (being a man or woman) but also blur the distinctions between sexuality and gender (Goldberg et al. 2020). Sexuality and gender are no longer distinctly singular axes of identity.

Queer and genderqueer are contemporary examples of identity labels that eschew binary sexuality and gender categories, blurring, combining, or merging conceptualizations of sexuality and gender in identity, or rejecting notions of discrete categories of or for sexuality and gender (Hammack et al. 2022, Watson et al. 2020). For example, a recent study reported that nearly one in four sexual minority young people describe their identity with a label besides lesbian, gay, or bisexual (Watson et al. 2020) and that the use of emerging labels varied by gender and ethnoracial identity. Furthermore, cisgender girls with multiple ethnoracial identities were more likely to identify with an emerging sexual identity label than those with multiple ethnoracial identities who were another gender. In a qualitative investigation of personal meanings of sexual identities, queer adults described their attractions to multiple and diverse gender expressions and identities (attraction “regardless of their gender”) as a central component of a queer identity (Galupo et al. 2017). Although queer and genderqueer are two contemporary expressions of identities for which gender and sexuality are inseparable, notably, two-spirit is an earlier example of identities that were expressed in distinctive and diverse ways across indigenous groups in North America (Davis 2019) and existed well before Western conceptualizations and distinctions of sexuality and gender (Carrier et al. 2020, Robinson 2020, Sheppard & Mayo 2013). Thus, although language and accessibility to information regarding diverse identities have expanded, it is important to note that the identities that such labels represent are by no means new.

### Identity Complexities and Intersectionality

Beyond the intertwined nature of sexuality and gender, intersectional perspectives illuminate that sexual identity has never been a singular domain or axis of identity but has always been deeply intertwined with—or inseparable from—other core personal identity components that historically have been treated as distinct or separate. The complexities of sexual identity (and the intersection of sexual and gender identities) intersect with racial, social class, religious, disability, social, and personal identities (see the sidebar titled Intersectional Stigma for a discussion of the adjacent literature on intersectional stigma). Research in the past decade has made more visible the diverse experiences and meanings of sexual identities at these intersections (Natl. Acad. Sci. Eng. Med. 2020).

Multiple scholars have investigated identities at the intersection of minority sexuality and racialized/ethnic identities. For example, “same-gender loving” is an identity and label for some African American disadvantaged youth, who are less likely to identify with the “gay” label compared with White youth (Parks 2001). A study of Black men who have sex with men (MSM) in the Deep South identified multiple, and at times overlapping, meanings of MSM as sexual behavior as well as identity for some men. Through focus groups, the study revealed internalization of categories of sexual behaviors, as well as community-embedded identities unique to the Deep South Black community, characterized by intersections of race, socioeconomic status, sexual behavior, and the community context (Truong et al. 2016). A study of gay Asian men provides an example of a group for whom the stigma associated with both their sexual and racialized/ethnic identities becomes a basis for collective identity—as “Gaysian”—as well as for collective stigma management strategies with family and friends to redefine and destigmatize homosexuality and maintain harmony in families and relationships (Han et al. 2014).

Other work gives attention to religious identity and its intersections with sexual identities. In a study of the role of religion in the lives and relationships of Jewish lesbians, participants characterized their experience as living as a “double minority” and described living in inclusive climates as integral to integration of religious and sexual identities (Barrow & Kuvalanka 2011). A study of Black, same-gender loving, Christian men described the ways that these men reconcile or integrate their religious, racialized/ethnic, and sexual identities (Lassiter 2015).

### Sexual Identities, Human Development, and the Life Course

Certainly, personal identities are salient across the life course. Yet late childhood and adolescence have long been understood as the crucial and primary period for identity development (Erikson 1968), and in the past decade, there has been an expansion of human developmental (ontological) understandings of the development of sexual identities (Bishop et al. 2020). The biopsychosocial changes associated with puberty prompt normative development and, for youth who develop awareness and identity as sexual minorities, a simultaneous emerging consciousness of that minority identity status and its personal and social meanings (Natl. Acad. Sci. Eng. Med. 2020). Contemporary youth are confronting and problematizing binaries of sexuality and gender (for example, challenging the ways researchers define and measure sexual and gender identities) (Watson et al. 2020)—that is, it has been young people who have been most adaptive and responsive to expanding notions of LGBTQ+.

The developmentally, socially, and historically distinctive feature that has emerged in recent decades is that people—in particular, adolescents—are afforded possibilities to consider or question the complexities of their sexualities. Not only have new identity categories and labels emerged, but so have new intersectional understandings of sexualities with respect to identity. There are more numerous and diverse voices articulating identity meanings and labels, expanding far beyond the historically traditional or typical sexual identities rooted in the binaries of sexuality (straight or gay), sex, and gender (being a man or woman, masculine or feminine; Watson et al. 2020).

In addition to expanding definitions and meanings, another emergent possibility is that liminality has become understood by some as both a process of sexual identity exploration and also as a possibility for identity itself, as is the case for people who identify as “questioning” or “unsure” (Katz-Wise 2015, Savin-Williams 2017, Williams et al. 2003; see the sidebar titled Can Sexual Orientation Be Changed?). Sometimes termed “fluidity,” the idea of liminal, developing, or changing sexual identity has emerged as an identity space, particularly in the context of adolescent development. On the one hand, this acknowledgment of identity development is fully consistent with understandings of the adolescent period of identity development. Yet, on the other hand, those conceptualizations have been linear, presuming that the outcome of exploration is identity consolidation or achievement (Cass 1979, Troiden 1989). Contemporary understandings that allow for fluidity or liminality challenge the presumed linearity, or resolution, of identity processes, as well as the assumption that this liminality is confined to adolescence.

### Relational Identities

In the sense that identities are socially constructed, they develop personal and social meaning in interaction with others. In one of the first investigations to use a national probability sample to examine the demographic characteristics of people who identify as queer (or genderqueer), queer people were more likely to report attractions to and relationships with queer or gender nonbinary people (Goldberg et al. 2020). These patterns illuminate the ways that sexual and gender identities shape and are intertwined with relationships and partnering: Identities can be understood as relational.

The life course concept of interdependent lives points to the relevance of relationships for marking space and meaning in individuals’ lives and offers the possibility for (sexual) identities rooted in understandings of the self that are defined by interdependence with primary relationships. A recent article documented the ways that some sexual minority people named their relationships when describing their identities (e.g., partner, husband, wife) (Mernitz et al. 2022). Notably, in describing identities in terms of their relationship, sexual minority people consistently described them in intersectional terms. Relationships were central to personal identities when they involved the negotiation of differences across gender expression or differences in race or ethnicity. For example, relationships were central to identity when couples negotiated racial differences, as in cases of mixed-race couples for whom the person of color had experiences navigating the world that were distinct from or not understood by their White partner. In such cases, racial or ethnic identity was entwined with the experience of the relationship, and the relationship itself was a salient component of self-identity.

Other work documents sexual identities that become defined in relational context. Demisexual, for example, is a sexual identity that applies when a person feels sexual attraction only in the context of emotional connection (Copulsky & Hammack 2021). Furthermore, there are multiple examples of sexual identities that are intertwined with community or subcultural meanings for interpersonal or sexual relationships and practices. Wilson (2009) describes “pillow princesses” as Black femme lesbian receivers of sexual penetration and pleasure, an identity for which meaning is rooted at the intersection of race, gender, sexuality, and sexual pleasure practices. Here, a sexual identity is understood in sociohistorical and community contexts as well as in terms of interpersonal relationships and practices—in relational terms.

Multiple studies have focused on “top” and “bottom” sexual position labels among gay men, particularly in the area of studies of HIV risk. In a study of US urban gay men, half labeled themselves as “top” or “bottom,” and these labels corresponded with sexual practices and preferences, as well as with HIV risk (gay men who identified as “bottoms” were more likely to be HIV positive) (Wegesin & Meyer-Bahlburg 2000). A study of South African gay men investigated ways that men construct identities and negotiate relationships (Henderson 2018), documenting that the “bottom” identity label can reflect stereotyped sexual roles while also powerfully resisting heteronormativity.

These studies point out that understanding sexual identities in terms of relationships includes understanding partnering and the identity of being in a relationship, the relational context of identities, and sexual practices as salient for sexual identity for many people. Hammack et al. (2019) have recently proposed a new paradigm of queer intimacy for understanding sexual and gender diversity and relationships that accounts for life course diversity in gender and sexual identities, incorporating relational power, sexual or romantic desire, chosen or biological family forms, and forms yet unknown.

## IMPLICATIONS FOR THE SOCIOLOGY OF SEXUALITIES AND SEXUAL MINORITIES

Recent decades have seen expanding understandings of sexual identity and growing diversity in the identities and labels that people use to name their identities and experiences. More than a decade ago, there was academic debate about whether sexual minority (“gay”) identities had become meaningless, particularly for youth (Russell et al. 2009, Savin-Williams 2009). Looking back on those debates today, we can see that the issue was not so much that “gay” no longer has meaning, but rather that the historically acknowledged sexual minority identities (gay, lesbian, bisexual) have been and are being reimagined and redefined—by and from an increasingly diverse and growing number of people and perspectives. What we witness now is the expanding diversity of sexual identities. At the same time, while the expressions of identity diversity are visible in new ways, sexual identity diversity has always existed—what is new is cultural, institutional, and personal acknowledgment of this diversity.

We have argued that these changes are socially constructed in ways that are fundamentally intersectional and shaped by life course forces that involve both individual human developmental change and the dynamics of relationships. There are multiple implications for the sociological study of sexual identities and sexualities. We have described ways that intersectional, life course approaches highlight multiple, diverse, and emerging strategies to understand, conceptualize, and analyze intersectional identities. As understandings and classifications or measures of sexual minority identities shift across time and contexts, new opportunities and constraints for research are emerging. In the context of significant advances in the measurement of sexual identities in recent years (Natl. Acad. Sci. Eng. Med. 2022), the expansion of sexual identities compels new methods and measures. How can the range of fields of sociological and social sciences capture the complexities of sexual identity diversity? Diverse epistemological approaches offer possibilities for advancing understandings of contemporary identities, ranging from autoethnographic to demographic. Ultimately, historical identity label frameworks may simply be too limited to capture the changing community, interpersonal, and intraindividual meanings of contemporary sexual identities.

Recent methodology innovations have answered long-standing critiques of quantitative approaches to studying intersectionality and identities (Bauer et al. 2021). For example, in a recent study that applied chi-squared automatic interaction detection (CHAID) analysis, the prevalence of substance use among youth was examined at the intersection of race/ethnicity, sexual orientation, gender identity, and sex assigned at birth. The CHAID method uses tree-based algorithms to identify combinations of discrete identities that have the strongest prevalence of a dichotomous dependent variable based on iterations of chi-squared tests of difference. The results illuminated groups defined by intersecting social identities for which youth had a particularly high (or low) burden of substance use: Transgender and gender diverse Latinx/o/a youth, particularly those assigned male at birth, were in high-prevalence groups for multiple indicators of substance use (Eisenberg et al. 2022).

One area for deeper intersectional and methodological inquiry is change and time. We have highlighted the role of life course and developmental change in understanding expanding sexual identities: Labels are static, but people are not. In their guidance for intersectional quantitative research, Else-Quest & Hyde (2016a,b) argue that intersectional research must treat identity as consisting of properties of both the individual and their social worlds—both of which change over time. As such, some have argued that intersectional research must capture this change with prospective and longitudinal methods (Ghavami et al. 2016, Mallory & Russell 2021). Whether and how to incorporate time as an intersectional category is a ripe area for future intersectional work.

Finally, at the root of sexual identity has been the dynamic interplay of sexualities, identities, and stigma. Attention to sexual identities has demanded attention to stigma and discrimination in the lives of sexual minority people. The greater intersectional awareness and understanding of expanding notions of LGBTQ+ offer not only a richer understanding of sexual minority people and their lives but also the possibility for deeper intersectional and coalitional approaches to understanding and confronting stigma and discrimination.

## Figures and Tables

**Table 1:** Glossary of terms

Term	Definition
**Cisnormativity**	The assumption that the vast majority of people are cisgender (i.e., that a person’s assigned sex is aligned with their gender identity). Embedded in cisnormativity is the implication that additional genders are other, abnormal, or deviant. As such, when cisnormativity structures social, political, and economic organization, it privileges those who are cisgender.
**Gender identity**	A person’s psychological or intrapersonal self-understanding as being a man or woman, a blend of genders, neither gender, or another gender. Gender identity can refer to how a person perceives themself and/or what they call themself. A person’s gender identity can be aligned with their sex assigned at birth (i.e., cisgender) or distinct from their sex assigned at birth (i.e., transgender). Gender identity does not imply a specific sexual orientation, and as such, both transgender and cisgender people can identify as straight, lesbian, gay, bisexual, and any other sexual identity.
**Genderqueer**	As an umbrella term, genderqueer identities are those that trouble binary gender boundaries between men and women. Genderqueer identities can include any gender identities that are distinct from man and woman, such as nonbinary, agender, genderfluid, and genderqueer, among others.
**Heteronormativity**	The assumption that the overwhelming majority of sexual attractions, behaviors, and relationships are heterosexual. Heteronormativity is the prevailing sexual model in the Western world, and it structures ideological, political, and economic social norms around gender and sexuality. As such, heteronormativity privileges those who are heterosexual and alienates those with nonheterosexual sexualities.
**Intersectionality theory**	Originating from late-twentieth-century Black feminist movements, intersectionality theory describes the means by which numerous and interlocking forms of structural privilege and oppression, including sexism, heteronormativity, cisnormativity, racism, and classism, intertwine to influence those who hold multiple marginalized identities across race, gender, class, and sexual identity, among others. Increasingly, scholars of LGBTQ+ people acknowledge that LGBTQ+ identities are inherently intersectional and must be represented as such.
**Queer (identity)**	Queer identity frequently refers to those whose gender and/or sexual identity, attractions, or behaviors fall outside of unchanging and binary assumptions. Many who identify as queer indicate a refusal to define their sexuality in relation to binary and stable notions of gender/sexuality; rather, they are explicitly naming the inclusion of those who fall outside of binary standards into their sphere of sexual and romantic attraction. Others who identify as queer are indicating a political alignment with the rejection of heteronormative and cisnormative structural prejudice. Some who are queer describe the importance of reclaiming a once-pejorative term. Finally, many who are queer are blurring the boundaries of sexual identity and gender identity labels, which have traditionally been understood as distinct social categories. As such, queer is used both as an umbrella term to describe those with minoritized sexual and gender identity, and as an individual identity that can allude to both and/or either sexual identity or gender identity.
**Sex (i.e., sex assigned at birth)**	A label specified during or before birth based on an infant’s physiological characteristics, such as their genitals, hormones, and chromosomes. Sex labels include male, female, and intersex.
**Sexual identity**	A person’s self-concept based on sexual and romantic attractions and/or behaviors, and/or a person’s societal identities with respect to a felt affiliation with or membership in a social group based on sexual orientation. Common societal categories for sexual identity include straight, lesbian, gay, bisexual, and queer, among others. Historically, sexual identity was indicative of one’s own and/or partner’s sex or gender; however, increasingly and especially for younger generations, notions of sexual identity as contingent on gender are changing.
**Sexual minority identity**	Identification (either personal or social) with a sexual identity that falls outside of heterosexuality. Common sexual minority identities include gay, lesbian, bisexual, queer, and pansexual, among others. Given that heteronormativity is prevalent in Western culture, sexual minority people have often been disenfranchised and underrepresented in social science.

## INTERSECTIONAL STIGMA

A body of research adjacent to intersectional identities explores intersectional stigma (Berger 2022, Natl. Acad. Sci. Eng. Med. 2020, Turan et al. 2019). In this work, axes of oppression operate not only with respect to personal identities but also through the stigma that a person may experience based on membership in a stigmatized group. Such work is intertwined with understanding personal identities, given that personal identities, like sexual identities, may be aligned with groups that have been stigmatized. Furthermore, one does not have to be part of a stigmatized group to experience stigma: For example, both sexual minority and majority people experience sexual prejudice (Parent et al. 2020). Researchers have begun to apply an intersectional stigma lens to understand intersections of identities and stigma. For example, a study of sexual minority youth of color investigated intersecting experiences of racial discrimination and LGB victimization, showing that the experience of both forms of discrimination had a compounding negative effect on mental health (Mallory & Russell 2021).

## CAN SEXUAL ORIENTATION BE CHANGED?

In the context of contemporary discourse and political debates regarding sexual orientation change therapies (i.e., “conversion” or “reparative” therapies), it is critical to acknowledge that “fluidity” does not mean external malleability but simply intraindividual identity development or change. There is no evidence that external efforts to change sexual identity have the intended effects; in fact, they have been shown to strongly threaten mental health (Blosnich et al. 2020, Green et al. 2020, Jacob 2015). Despite this, less than half of adult LGBTQ+ people in the United States reside in states that ban conversion therapy for minors (Mov. Adv. Proj. 2022).

## SUMMARY POINTS

1. Meanings and labels of sexual identity have been expanding.
2. A growing number of people identify as part of the LGBTQ+ umbrella, outside historically privileged and dominant sexual identities.
3. Sexual identities emerge through interpersonal and community interaction and shared experiences.
4. Sexual identity is a singular domain or axis of identity but is intertwined with—and inseparable from—other core personal identities: race, social class, and religion.
5. Sexual identities are situated historically, ontologically, developmentally, and interpersonally.

## FUTURE ISSUES

1. What are the best strategies for empirically measuring expanding sexual identities?
2. Beyond new categorizations of sexual minority people, how do expanding identities relate to intersectional systems of power and privilege?
3. How can the documentation of expanding sexual identities contribute to coalition building in response to intersectional stigma, discrimination, and oppression?
4. What role can interdisciplinary scholarship play in better understanding and documenting expanding sexual identities?
